# Assessment of the intracellular distribution of copper in liver specimens from cats

**DOI:** 10.1371/journal.pone.0264003

**Published:** 2022-02-14

**Authors:** Punyamanee Yamkate, Randi M. Gold, David C. Twedt, Jan S. Suchodolski, Joerg M. Steiner, Jonathan A. Lidbury

**Affiliations:** 1 Gastrointestinal Laboratory, Department of Small Animal Clinical Sciences, Texas A&M University, College Station, Texas, United States of America; 2 Department of Clinical Sciences, College of Veterinary Medicine and Biomedical Sciences, Colorado State University, Fort Collins, Colorado, United States of America; University of Life Sciences in Lublin, POLAND

## Abstract

The intracellular distribution of copper in the liver has been investigated in dogs and humans. However, this has not been reported in cats. This study aimed to assess the intracellular copper distribution in liver specimens from cats with a range of hepatic copper concentrations. Twenty-nine frozen liver specimens from cats were included. Each liver specimen was divided into two pieces for overall copper quantification and tissue fractionation. The copper concentrations in liver specimens and liver fractions were measured by flame atomic absorption spectroscopy. Five specimens had copper concentrations < 100 μg/g dry weight, eight had copper concentrations between 100 and 180 μg/g, 14 had copper concentrations between 181 and 700 μg/g, and two had copper concentrations >700 μg/g. Only one specimen had positive copper staining. Regardless of the overall concentrations, copper was mostly found in the cytosolic fraction followed by the nuclear, large granule, and microsomal fractions. Our findings indicate that similarly to other species, intracellular copper is predominantly found in the cytosolic and nuclear fractions in cats. The distribution in cats with copper-loaded conditions, such as primary copper hepatopathy, was not assessed but warrants evaluation.

## Introduction

Copper is an essential trace element that serves as a cofactor for several enzymes that are required for growth, development, and maintenance of living cells [1, 2]. The liver plays a major role in copper metabolism and homeostasis. Dietary copper is absorbed from the small intestines and enters hepatocytes from where it is either distributed throughout the body in protein-bound forms, or eliminated through biliary excretion if there is an excess of copper [3]. Copper homeostasis is tightly regulated since excess copper can become a potentially toxic transition metal and generate reactive oxygen species (ROS) [2]. Excessive ROS promotes oxidative stress and consequently induces hepatic necrosis and inflammation with possible subsequent fibrosis [4]. Liver injury caused by copper, known as copper-associated hepatopathy, can eventually progress to hepatic cirrhosis and failure [5]. Cats with primary copper-associated hepatopathy (PCH) have been reported to have copper concentrations > 700 μg/g (range: 704–7,041 μg/g) dry weight liver [6]. The copper accumulation was found to be localized in centrilobular areas associated with most of the hepatocellular histological changes [6–8]. Additionally, excessive copper accumulation is reported to occur in cats secondary to other cholestatic hepatobiliary diseases. Hepatic copper concentrations in these cats were reported to be lower than those with PCH and the accumulation was localized predominately to the periportal regions [6].

Measurement of copper in subcellular liver fractions may provide an important insight into the pathogenesis of copper-associated hepatopathy. Subcellular organelles, including those found in the liver, can be separated by differential centrifugation, a technique for crude separation and purification of organelles and other particles based on their sedimentation rate which depends upon their size [9]. Using this technique, liver homogenate can be separated into four main compartments: the nuclear, large granule, microsomal, and cytosol fractions [9]. The nuclear fraction contains the nuclei of hepatocytes. However, since the differential centrifugation only separates organelles crudely, the nuclear fraction also contains cytoskeleton, plasma membrane, and intact hepatocytes. The large granule fraction contains mitochondria, lysosomes, and peroxisomes, whereas the microsomal fraction contains microsomes and other small vesicles. Lastly, the cytosolic fraction contains an intracellular fluid of cytoplasm, ribosomes, large macromolecules, and other soluble proteins [10].

There are many studies regarding the intracellular distribution of hepatic copper in mammals. In healthy control rats, copper was mainly found in the cytosolic fraction [11, 12]. In contrast, in copper-loaded rats, the copper was mainly localized in the large granule fraction [11]. In healthy adult humans, the proportion of copper was highest in the cytosol, whereas, patients with Wilson’s disease (WD), a genetic disorder associated with excessive hepatic copper accumulation, had a greater proportion of hepatic copper in the large granule fraction [13]. In Bedlington Terriers with copper-associated hepatopathy, an ultrastructural study using an electron microscope found that copper rapidly accumulated in liver lysosomes after copper infusion into the intestinal lumen. In contrast, there was only a small number of copper granules in bile canaliculi [14]. The cumulative data from the above studies suggested that under physiological conditions, copper is mainly found in the cytosolic compartment of hepatocytes. When rats, humans, and dogs are loaded with copper experimentally or due to spontaneously occurring defects in copper excretion, copper becomes distributed into the other compartments, particularly the large granule fraction [11–14]. However, to date, the intracellular distribution of copper in the liver of cats has not been reported.

The objective of this study was to describe the intracellular distribution of copper in liver specimens from cats with a range of copper concentrations.

## Materials and methods

### Sample collection

Twenty-nine frozen liver tissue specimens from cats were included in this study. The specimens had been collected postmortem from feral cats that were euthanized in Malaysia in 2017 for non-study related reasons. Hepatic wedge biopsy specimens (approximately 2 x 1 x 1 cm in size) were immediately placed on ice for 1–2 hours, and then frozen at –80°C. In 2017, portions of these specimens had been sectioned and were processed using routine histology techniques for histopathological evaluation and qualitative copper assessment. The remainder of these specimens were kept frozen at –80°C until further analysis was performed in 2019. This work did not involve the use of live animals, and therefore, institutional animal care and use committee approval was not required.

### Histopathological analysis and hepatic qualitative copper assessment

The formalin-fixed paraffin-embedded tissue blocks were cut at 4-μm thickness. Tissue sections were stained with hematoxylin and eosin (H&E) for histopathological analysis. Tissue specimens were classified based on previously published criteria of inflammation, lipid accumulation, and the presence of neoplastic cells [15]. The scoring system and classification criteria are described in S1 and S2 Tables. Tissue sections were also stained with rhodanine for qualitative copper assessment using a modified scoring system adapted from copper staining criteria used in dogs (Table 1) [16]. All tissue sections were analyzed by a board-certified veterinary anatomic pathologist in a blinded fashion (RMG).

**Table 1:** A copper scoring system for qualitative copper assessment modified from a scoring system used for liver biopsy specimens in dogs [16].

Score	Accumulation of copper granules in hepatocytes or macrophages
0	No copper granule accumulation
1	Variable copper granules in an occasional hepatocyte
2	Small to moderate numbers of copper granules in < 50% of hepatocytes
3	Moderate to large numbers of copper granules in 50–75% of hepatocytes; copper-containing macrophages may be present
4	Moderate to large numbers of copper granules in > 75% of hepatocytes; copper-containing macrophages may be present
5	Panlobular presence of copper granules, usually associated with copper-containing macrophages

### Tissue fractionation

Each remaining liver specimen was divided into two pieces. One (at least 50 mg wet weight) was used for overall copper quantification and the second (approximately 1 g wet weight) was processed for fractionation. The samples used for fractionation were individually suspended and homogenized in 0.25 M sucrose, pH 8 in a 1:6 (w:v) ratio at 4°C. The differential centrifugation protocol for tissue fractionation has previously been described [10]. Briefly, the liver homogenate was centrifuged (Centrifuge 5810 R; Eppendorf, Hamburg, Germany) in a fixed-angle rotor (Rotor F-34-6-38; Eppendorf) at 600 × g for 10 minutes to separate the nuclear pellet, and at 8,500 × g for 12 minutes to separate the large granule pellet. Then the supernatant was centrifuged (Optima MAX-XP; Beckman Coulter, Brea, CA) with a fixed-angle rotor (MLA-55; Beckman Coulter) at 105,000 × g for 60 minutes to separate the microsomal pellet. The remaining supernatant was considered the cytosolic fraction.

The fractionation procedure was validated with the enzymatic activity assays and DNA quantitation. The assays were performed in all four fractions from five frozen liver specimens. These specimens were collected and stored under the same conditions and similar storage time as the 29 specimens in the study. A DNA assay and activity assays for glutamate dehydrogenase, glucose 6 phosphatase, and alanine aminotransferase assays were used to confirm the nuclear, large granule, microsomal, and cytosolic fractions, respectively [17–19]. Each assay was performed according to the manufacturer’s instructions. Briefly, DNA fluorometric assay (QFDN-250; BioAssay Systems, Hayward, CA; 1:400 dilution) was determined at 360/460 nm (excitation/emission) using a microplate reader (BioTek Synergy 2; BioTek, Winooski, VT). Glucose 6 phosphatase colorimetric activity (E-120; Biomedical Research Service, Buffalo, NY; 1:100 dilution) was detected at an absorbance OD 620 nm with a microplate reader (BioTek Synergy 2; BioTek). Glutamate dehydrogenase activity (C550-0A; Catachem Inc., Oxford, CT; 1:10 dilution), and alanine aminotransferase assays (ORS6107; Beckman Coulter; 1:10 dilution) were analyzed using the chemistry analyzer (DxC 700 AU; Beckman Coulter).

### Hepatic copper quantification

All liver specimens and liver fractions were submitted to the Veterinary Diagnostic Laboratories at Colorado State University for copper quantification by flame atomic absorption spectroscopy (FAAS). FAAS is simple to perform with good precision for single-element measurement [20]. In this laboratory, FAAS has been validated for a minimum sample size of 50 mg wet weight. This technique dissociates the copper in tissue samples into atoms using a flame. Copper atoms absorb the light from the lamp and the absorbance is measured [20]. Then, the concentration is calculated in the unit of μg/g wet weight. The wet weight concentration is then multiplied by 4 to be converted to approximate dry weight liver tissue [21]. Based on this laboratory’s reference interval, copper concentrations < 100 μg/g dry weight liver were considered deficient level and the concentrations < 180 μg/g dry weight liver (i.e., the reported upper limit of the reference interval for cats) [21] were considered normal for the purpose of this study. The concentrations > 700 μg/g dry weight liver were reported as the speculated threshold for PCH cats [6]. The specimens in our study were stratified into four groups based on overall hepatic copper concentrations: specimens with copper concentrations < 100 μg/g (Group 1), concentrations between 100–180 μg/g (Group 2), concentrations between 181–700 μg/g (Group 3), and concentrations > 700 μg/g dry weight (Group 4).

### Statistical analysis

Statistical analysis was performed using a commercially available statistical software package (JMP Pro version 14; SAS Institute, Cary, NC). The Shapiro–Wilk W test was used to determine the normality of all data sets. Data were reported as median (minimum–maximum) values. DNA concentration and enzymatic activities were compared among four liver fractions using the Kruskal-Wallis test and followed by Dunn’s multiple comparison test. The copper distribution in liver fractions was calculated as a percentage of total copper. Then, the distribution in each fraction was compared among liver specimens from cats with a different range of copper concentrations using the Kruskal-Wallis test. The relationship between the intracellular distribution of copper and overall hepatic copper concentrations was also assessed using Spearman’s rank correlation. Statistical significance was set at p < 0.05.

## Results

### Validation of subcellular fractionation

The DNA and enzymatic activity assays are reported in Table 2. The DNA concentration among four fractions was significantly different (p = 0.006). The concentrations in nuclear fraction were found significantly higher than large granule, microsomal, and cytosolic fractions (p = 0.02, 0.04, and 0.02, respectively). The highest activity of glutamate dehydrogenase was detected in the large granule fraction followed by the nuclear fraction; however, no significant difference in activity of this enzyme was found between fractions (p = 0.07). Glucose 6 phosphatase activity was highest in the microsomal fraction, but there were also similar activities in the nuclear and large granule fractions. The activity in the microsomal fraction was significantly higher than the activity in the cytosolic fraction (p = 0.01). Lastly, the cytosolic fraction had the highest activity of alanine aminotransferase, but a significant difference in this enzyme activity was not found between fractions (p = 0.27).

**Table 2:** Summary of the DNA and enzymatic activity assays in four liver fractions from five frozen cat liver specimens.

	DNA concentration (ng/mL)	Glutamate dehydrogenase activity (U/L)	Glucose 6 phosphatase activity (OD)	Alanine aminotransferase activity (U/L)
Nuclear fraction	258,993*,**	6,468	0.13	4,147
	(201,439–690,648)	(4,825–19,518)	(0.07–0.24)	(790–7,512)
Large granule fraction	<100**	9,594	0.15	5,607
	(<100^a^–201,439)	(4,050–23,602)	(0.08–0.19)	(695–11,320)
Microsomal fraction	<100*	57	0.16***	6,646
	(<100–230,216)	(2–15,777)	(0.1–0.46)	(1,021–13,034)
Cytosolic fraction	<100**	2,668	0.05***	13,243
	(<100–143,885)	(1,278–6,057)	(0.04–0.08)	(1,895–18,326)

The data is reported as the median (minimum–maximum).

^a^The lower limit of the detection of the assay DNA assay is 100 ng/mL.

The DNA concentration in nuclear fraction was significantly higher than other fractions (*p = 0.04, **p = 0.02), The Glucose 6 phosphatase activity was significantly higher in the microsomal than the cytosolic fraction (***p = 0.01).

### Histopathological analysis and hepatic copper assessment

Liver tissues from 29 feral cats, 20 males and nine females were evaluated. The estimated age of the cats ranged from 1 to 7 years. On histopathological evaluation, 25 specimens had no significant histologic hepatic changes, two specimens had hepatic steatosis, and two specimens had hepatic inflammation. One liver specimen stained positive for rhodanine. The copper accumulated in the centrilobular and midzonal areas and was given a score of 2 out of 5. This specimen had no significant histologic hepatic changes and the hepatic copper concentration was 728 μg/g dry weight. The data of individual estimated age, sex, histopathological analysis, and overall hepatic copper concentrations of the specimens in this study are available in S3 and S4 Tables.

### Intracellular distribution of copper in liver fractions

The 29 specimens were stratified into four groups based on hepatic copper concentrations. Five specimens had copper concentrations < 100 μg/g dry weight (Group 1), eight had copper concentrations between 100–180 μg/g (Group 2), 14 had copper concentrations between 181–700 μg/g (Group 3), and two had copper concentrations > 700 μg/g (Group 4). The copper concentration in the fractions of hepatic tissue was reported as a percentage of the total copper concentration (Table 3). There was no significant difference in estimated age or sex among these 4 groups of specimens (p = 0.56, and p = 0.21, respectively).

**Table 3:** Summary of the intracellular distribution of copper in liver specimens from cats with copper concentrations < 100 μg/g dry weight (Group 1; n = 5), 100–180 μg/g dry weight (Group 2; n = 8), 181–700 μg/g dry weight (Group 3; n = 14), > 700 μg/g dry weight (Group 4; n = 2).

	Copper < 100 μg/g dry weight (Group 1)	Copper 100–180 μg/g dry weight (Group 2)	Copper 181–700 μg/g dry weight (Group 3)	Copper > 700 μg/g dry weight (Group 4)
Nuclear fraction	27%	19%	19%	25%
	(11–42%)	(11–24%)	(9–40%)	(22–28%)
Large granule fraction	12%	13%	17%	23%
	(9–34%)	(9–28%)	(3–23%)	(19–26%)
Microsomal fraction	2%	6%	7%	7%
	(1–15%)	(2–9%)	(1–16%)	(5–10%)
Cytosolic fraction	52%	62%	58%	45%
	(40–64%)	(57–66%)	(37–79%)	(42–48%)

The distribution in each fraction is reported as the median (minimum–maximum) percentage.

**Fig 1:**
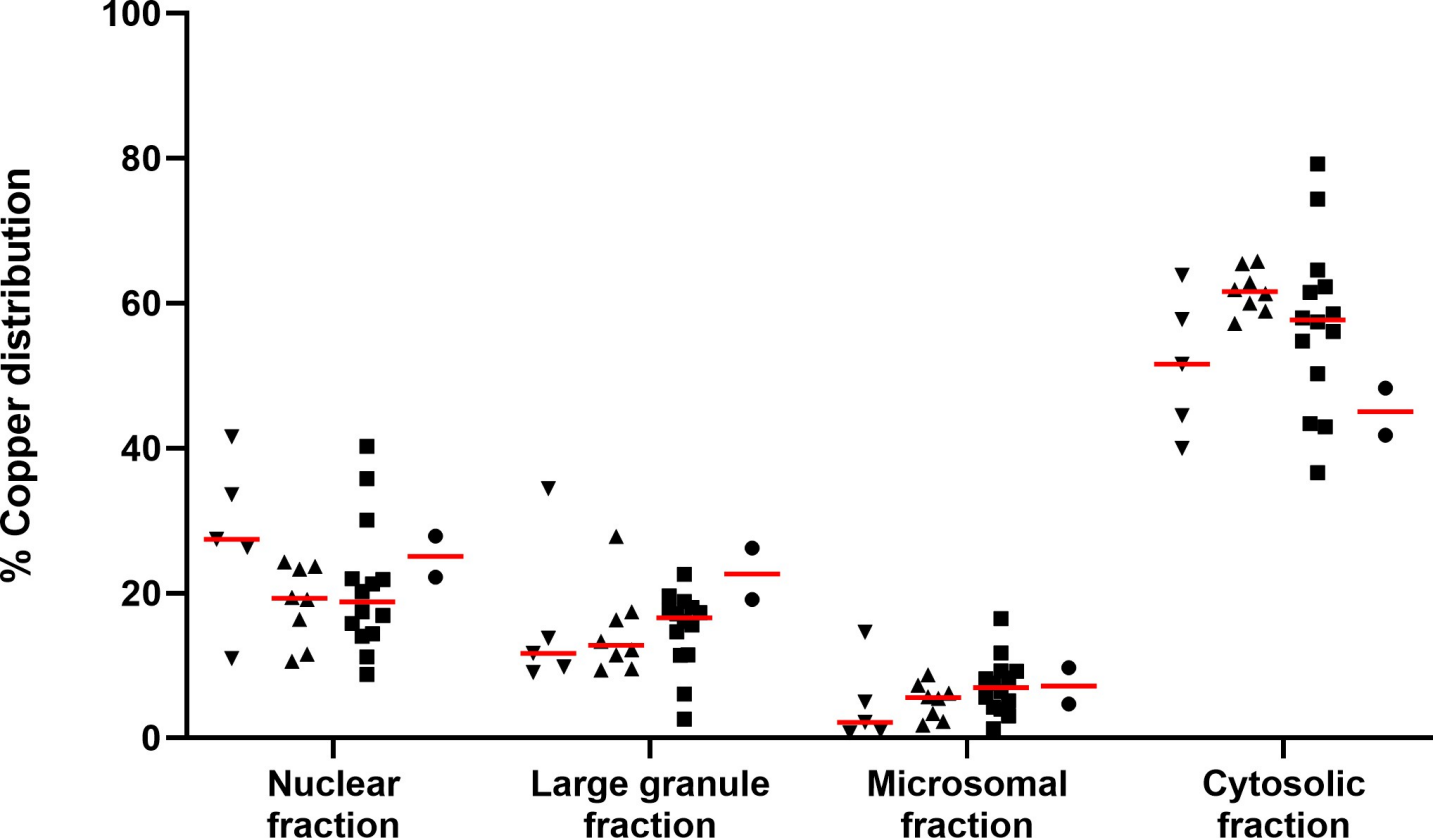
The intracellular distribution of copper in liver specimens from cats. (▼) specimens with copper concentrations < 100 μg/g dry weight (Group 1; n = 5), (▲) specimens with copper concentrations 100–180 μg/g dry weight (Group 2; n = 8), (■) specimens with copper concentrations 181–700 μg/g dry weight (Group 3; n = 14), (●) specimens with copper concentrations > 700 μg/g dry weight (Group 4; n = 2). In all 4 groups, copper was found most abundantly in the cytosolic fraction, followed by the nuclear, large granule, and microsomal fractions, respectively. The red lines represent the medians.

**Fig 2:**
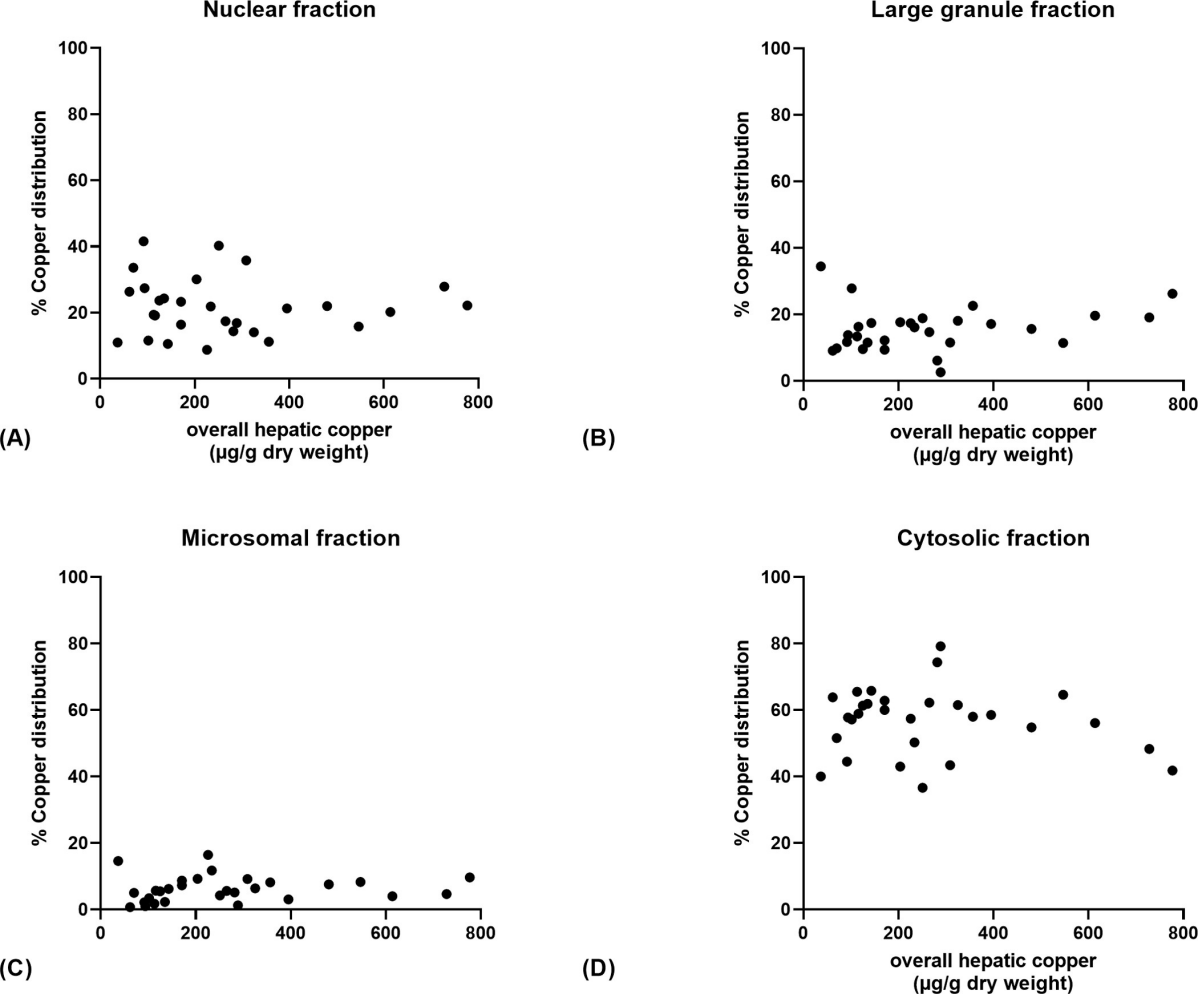
The correlation between the intracellular distribution of copper and hepatic copper concentrations. (A) nuclear fraction (*r*_*s*_ = -0.10, p = 0.60), (B) large granule fraction (*r*_*s*_ = 0.23, p = 0.22), (C) microsomal fraction (*r*_*s*_ = 0.28, p = 0.14), (D) cytosolic fraction (*r*_*s*_ = -0.06, p = 0.77). There was no correlation between the percentage of copper in any of the subcellular fractions and the overall hepatic copper concentrations.

Regardless of the hepatic copper concentrations, the highest intracellular copper fraction was cytosolic followed by the nuclear, large granule, and microsomal fractions, respectively (Fig 1). There was no significant difference in the intracellular distribution of copper for any of the four liver fractions among the four groups (p > 0.05 for each comparison). Additionally, Spearman rank’s correlation showed there was no significant relationship between the percentage of copper in any of the subcellular fractions and the overall hepatic copper concentration (p > 0.14; Fig 2).

## Discussion

We determined the intracellular distribution of copper in liver specimens from cats using a differential centrifugation technique to obtain four subcellular fractions. Copper was mainly found in the cytosolic fraction.

A DNA concentration assay was used to validate the nuclear fraction and enzymatic activities of glutamate dehydrogenase, glucose 6 phosphatase, and alanine aminotransferase were used to confirm large granule, microsomal, and cytosolic fractions, respectively [17–19]. DNA concentrations and enzymatic activities were found to be highest in their corresponding fractions, which provided some support for the effectiveness of this differential centrifugation technique in frozen cat liver. Similar results were obtained using 2 fresh liver specimens from cats (results not shown). However, lower DNA concentrations and enzymatic activities were also found in other subcellular fractions to varying degrees. It has previously shown that DNA is detected mainly in nuclei fraction, but also in a smaller amount in the extranuclear fractions [22]. Overlapping enzymatic activities were also reported in other studies. Glutamate dehydrogenase activity in rat liver tissue was found in the nuclear fraction in addition to the large granule fraction [23, 24]. Glucose 6 phosphatase activity was reported in the nuclear fraction as well as the microsomal fraction [24, 25]. Alanine transferase was mainly found in the cytoplasm but this enzyme can also be presented in other compartments, such as the mitochondria [26]. In addition, studies in a carnivorous bird, rats, and cats have used similar differential centrifugation protocols to separate liver fractions [27, 28]. In their studies, the liver was homogenized in sucrose solution (1:9 w:v ratio). The differential centrifugation was performed at 3 different speeds, which were 600 x g (10 min), 10,000 x g (12 min), and 105,000 x g (60 min). Taken together these findings support the validity of the fractionation protocol using centrifugation that we used; however, the results should be interpreted cautiously as the contamination between fractions likely occurred to some extent.

Regardless of the overall hepatic copper concentrations, copper was found to be most abundant in the cytosolic fraction, followed by the nuclear, large granule, and microsomal fractions. To our knowledge, this is the first time that the hepatic intracellular copper distribution has been reported in this species. Our findings are similar to those reported for other species, such as rats, dogs, and humans, with normal hepatic copper concentrations [11–13]. In this study, the intracellular distribution of copper in all 4 fractions was consistent over a range of total hepatic copper concentrations. Studies in other species in animals with copper-loaded conditions showed significantly higher proportions of copper in the large granule fraction, which contains lysosomes, mitochondria, and peroxisomes [10], when compared to other fractions [11, 13]. It was also shown that copper predominantly accumulated in lysosomes [14, 29]. However, our sample size was relatively small and only one specimen within the group of elevated copper above the ULRI showed positive rhodanine staining. In addition, the specimens were unlikely to have true copper-loaded conditions. Two specimens in our study had copper concentrations > 700 μg/g dry weight liver, the speculated threshold for PCH in cats [6]. However, considering the histopathological findings, none of them were considered to have PCH. Therefore, such a redistribution of hepatic copper cannot be ruled out in cats with copper-loaded conditions, such as PCH, as this group of cats was not represented in our study.

Fourteen of 16 specimens with copper concentrations above the ULRI did not have significant histopathological changes of the liver. The reason why cats without obvious hepatic histological lesions had copper concentrations greater than the ULRI is not known. One possibility is that the reference interval used is not appropriate for feral cats from Malaysia. Previous studies of hepatic copper concentrations in cats found a wide range of hepatic copper concentrations in healthy control cats and cats without hepatobiliary diseases (e.g., 9.5–451.6 μg/g dry weight liver in 23 cats and 10.8–394.8 μg/g dry weight liver in 47 cats) [30, 31]. We previously found copper concentrations greater than the ULRI in cat liver tissue submitted to a US-based veterinary laboratory even when hepatic lesions were not appreciated [15].

Our study is the first to describe the hepatocellular distribution of copper in cats but is subject to some limitations. The study population consisted of feral cats with no prior medical history. The sample group lacked cats having confirmed PCH, and higher hepatic copper concentrations than seen in most of the cats included in this study are likely required for positive rhodanine staining. Two cats with presumed PCH reported on in two different case reports that had positive copper staining were found to have extremely high hepatic copper concentrations (i.e., 4,074 and 4,170 μg/g dry weight liver respectively) [7, 8]. It is possible that the intracellular distribution of copper would have been different in cat livers with higher hepatic copper concentrations or those with PCH. Additionally, the information on the diet that may have an effect on hepatic copper accumulation in these cats was not available. Furthermore, the relatively small and unequal sample size could have led to a type II error, leading to failure to detect a difference in the intracellular distribution of copper between groups in this study. Thus, the evaluation regarding this issue in a greater number of cats, including some with PCH is needed. Although stored frozen tissue samples are reported to provide reproducible subcellular fractionation results [32], repeating the study using fresh liver samples may have shown a different subcellular copper distribution. Lastly, despite the fact that the protocol of differential centrifugation used in this study was similar to previous studies in a carnivorous bird, rats, and cats [27, 28], this technique provides crude separation of the organelles. Contamination between fractions likely occurred to some extent and so results should be interpreted cautiously.

Intracellular copper in liver specimens from cats with a range of overall copper concentrations was predominantly found in cytosolic and nuclear fractions. The distribution in cats with copper-loaded conditions, such as PCH, was not assessed but warrants evaluation.

## Supporting information

S1 Table Scoring system for histopathological classification. A modified system for liver specimens based on inflammatory cell accumulation and percentages of hepatocytes showing lipid accumulation.
(DOCX)

S2 Table Histopathological classification based on the scores for inflammation, lipid accumulation, and the presence of neoplastic cells. (DOCX)

S3 Table List of specimens with copper concentrations below the upper limit of the reference interval (n = 13). The information of individual estimated age, sex, histopathological analysis, and overall hepatic copper concentration.
(DOCX)

S4 Table List of specimens with copper concentrations above the upper limit of the reference interval (n = 16). The information of individual estimated age, sex, histopathological analysis, and overall hepatic copper concentration.
(DOCX)
