# Supplementary material for: Assessment of the intracellular distribution of copper in liver specimens from cats
Source: PLoS One. 2022 Feb 14;17(2):e0264003. doi: 10.1371/journal.pone.0264003 (PMC8843214; doi:10.1371/journal.pone.0264003)
Supplement: S1 Table — A modified system for liver specimens based on inflammatory cell accumulation and percentages of hepatocytes showing lipid accumulation. (DOCX) [file pone.0264003.s001.docx]

**S1 Table.** **Scoring system for histopathological classification.** A modified scoring system for liver specimens based on inflammatory cell accumulation [1] and percentages of hepatocytes showing lipid accumulation [2].

| **Score** | **Accumulation of inflammatory cells** |
| --- | --- |
| 0 | None |
| 1 | Mild |
| 2 | Mild to moderate |
| 3 | Moderate |
| 4 | Severe |
| **Score** | **Percentage of hepatocytes with lipid accumulation** |
| 0 | < 80% |
| 1 | ⩾ 80% |

**References**

1. Fragkou FC, Adamama‐Moraitou KK, Poutahidis T, Prassinos NN, Kritsepi‐Konstantinou M, Xenoulis PG, et al. Prevalence and clinicopathological features of triaditis in a prospective case series of symptomatic and asymptomatic cats. J Vet Intern Med. 2016;30(4):1031-45.

2. Center SA. Feline hepatic lipidosis. Vet Clin North Am Small Anim Pract. 2005;35(1):225-69.
