# Supplementary material for: Assessment of the intracellular distribution of copper in liver specimens from cats
Source: PLoS One. 2022 Feb 14;17(2):e0264003. doi: 10.1371/journal.pone.0264003 (PMC8843214; doi:10.1371/journal.pone.0264003)
Supplement: S2 Table — (DOCX) [file pone.0264003.s002.docx]

**S2 Table.** **Histopathological classification based on the scores for inflammation, lipid accumulation, and the presence of neoplastic cells [3].**

| Group | Scoring criteria |
| --- | --- |
| No significant histopathological hepatic changes | Inflammation 0–1; lipid accumulation 0 |
| Hepatic steatosis | Inflammation 0–2; lipid accumulation 1 |
| Hepatic inflammatory or infectious disease | Inflammation ⩾2; lipid accumulation 0 |
| Neoplasia | Presence of neoplastic cells, regardless of inflammatory and lipid accumulation scores |

**Reference**

3. Yamkate P, Gold RM, Xenoulis PG, Steiger K, Twedt DC, Suchodolski JS, et al. Assessment of copper accumulation in archived liver specimens from cats. J Feline Med Surg. 2021;23(6):526-33.
