# Supplementary material for: Assessment of the intracellular distribution of copper in liver specimens from cats
Source: PLoS One. 2022 Feb 14;17(2):e0264003. doi: 10.1371/journal.pone.0264003 (PMC8843214; doi:10.1371/journal.pone.0264003)
Supplement: S3 Table — The information of individual estimated age, sex, histopathological analysis, and overall hepatic copper concentration. (DOCX) [file pone.0264003.s003.docx]

**S3 Table.** **List of specimens with copper concentrations below the upper limit of the reference interval (n = 13).** The information of individual estimated age, sex, histopathological analysis and overall hepatic copper concentration.

| **Liver specimen** | **Histopathological analysis** | **Hepatic copper concentration (µg/g dry weight)** | **Copper distribution (%)** | | | | **Estimate age** | **Sex** |
| --- | --- | --- | --- | --- | --- | --- | --- | --- |
|  |  |  | **Nuclear** | **Large granule** | **Microsomal** | **Cytosolic** |  |  |
| Case 1 | No significant histopathological hepatic changes | 135 | 24 | 12 | 2 | 62 | 2-3 | Female |
| Case 2 | No significant histopathological hepatic changes | 70 | 34 | 10 | 5 | 52 | 1 | Male |
| Case 3 | Hepatic steatosis | 92 | 42 | 12 | 2 | 45 | 4-5 | Male |
| Case 4 | Hepatic inflammation | 171 | 23 | 9 | 7 | 60 | 2-3 | Male |
| Case 5 | No significant histopathological hepatic changes | 102 | 12 | 28 | 3 | 57 | 4-5 | Male |
| Case 6 | No significant histopathological hepatic changes | 171 | 16 | 12 | 9 | 63 | 1-2 | Male |
| Case 7 | No significant histopathological hepatic changes | 113 | 19 | 13 | 2 | 65 | 5-6 | Male |
| Case 8 | No significant histopathological hepatic changes | 94 | 27 | 14 | 1 | 58 | 2-3 | Male |
| Case 9 | No significant histopathological hepatic changes | 62 | 26 | 9 | 1 | 64 | 1-2 | Female |
| Case 10 | No significant histopathological hepatic changes | 116 | 19 | 16 | 6 | 59 | 6-7 | Male |
| Case 11 | No significant histopathological hepatic changes | 143 | 11 | 17 | 6 | 66 | 1-2 | Male |
| Case 12 | No significant histopathological hepatic changes | 125 | 24 | 10 | 5 | 61 | 1-2 | Female |
| Case 13 | No significant histopathological hepatic changes | 37 | 11 | 34 | 15 | 40 | 6-7 | Male |
