# Supplementary material for: Assessment of the intracellular distribution of copper in liver specimens from cats
Source: PLoS One. 2022 Feb 14;17(2):e0264003. doi: 10.1371/journal.pone.0264003 (PMC8843214; doi:10.1371/journal.pone.0264003)
Supplement: S4 Table — The information of individual estimated age, sex, histopathological analysis, and overall hepatic copper concentration. (DOCX) [file pone.0264003.s004.docx]

**S4 Table****.** **List of specimens with copper concentrations above the upper limit of the reference interval (n = 16).** The information of individual estimated age, sex, histopathological analysis and overall hepatic copper concentration.

| **Liver specimen** | **Histopathological analysis** | **Hepatic copper concentration (µg/g dry weight)** | **Copper distribution (%)** | | | | **Estimate age** | **Sex** |
| --- | --- | --- | --- | --- | --- | --- | --- | --- |
|  |  |  | **Nuclear** | **Large granule** | **Microsomal** | **Cytosolic** |  |  |
| Case 14 | Hepatic steatosis | 309 | 36 | 12 | 9 | 43 | 1-2 | Female |
| Case 15 | No significant histopathological hepatic changes | 614 | 20 | 20 | 4 | 56 | 2-3 | Male |
| Case 16 | No significant histopathological hepatic changes | 289 | 17 | 3 | 1 | 79 | 4-5 | Male |
| Case 17 | No significant histopathological hepatic changes | 357 | 11 | 23 | 8 | 58 | 3-5 | Female |
| Case 18 | No significant histopathological hepatic changes with positive copper staining (score of 2) | 728 | 28 | 19 | 5 | 48 | 6-7 | Female |
| Case 19 | No significant histopathological hepatic changes | 547 | 16 | 11 | 8 | 65 | 3 | Female |
| Case 20 | No significant histopathological hepatic changes | 777 | 22 | 26 | 10 | 42 | 3-5 | Male |
| Case 21 | No significant histopathological hepatic changes | 204 | 30 | 18 | 9 | 43 | 2-3 | Male |
| Case 22 | No significant histopathological hepatic changes | 325 | 14 | 18 | 6 | 62 | 1-2 | Female |
| Case 23 | No significant histopathological hepatic changes | 251 | 40 | 19 | 4 | 37 | 2-3 | Female |
| Case 24 | Hepatic inflammation | 395 | 21 | 17 | 3 | 59 | 2-3 | Male |
| Case 25 | No significant histopathological hepatic changes | 234 | 22 | 16 | 12 | 50 | 4-5 | Male |
| Case 26 | No significant histopathological hepatic changes | 282 | 14 | 6 | 5 | 74 | 2-3 | Male |
| Case 27 | No significant histopathological hepatic changes | 226 | 9 | 17 | 16 | 57 | 3-4 | Male |
| Case 28 | No significant histopathological hepatic changes | 265 | 17 | 15 | 6 | 62 | n/a | Male |
| Case 29 | No significant histopathological hepatic changes | 480 | 22 | 16 | 8 | 55 | 5 | Male |
